# Association between Gut Microbial Diversity and Carotid Intima-Media Thickness

**DOI:** 10.3390/medicina57030195

**Published:** 2021-02-25

**Authors:** Helga Szabo, Anita Hernyes, Marton Piroska, Balazs Ligeti, Peter Fussy, Luca Zoldi, Szonja Galyasz, Nora Makra, Dora Szabo, Adam Domonkos Tarnoki, David Laszlo Tarnoki

**Affiliations:** 1 Medical Imaging Centre, Semmelweis University, 1082 Budapest, Hungary; hernyes.anita@med.semmelweis-univ.hu (A.H.); piroskamarton94@gmail.com (M.P.); tarnoki2@gmail.com (A.D.T.); tarnoki4@gmail.com (D.L.T.); 2 Central Radiological Diagnostic Department, Medical Centre Hungarian Defence Forces, 1134 Budapest, Hungary; 3 Institute of Medical Microbiology, Semmelweis University, 1089 Budapest, Hungary; ligeti.balazs@itk.ppke.hu (B.L.); nora.makra@indamail.hu (N.M.); szabo.dora@med.semmelweis-univ.hu (D.S.); 4 Faculty of Information Technology and Bionics, Pazmany Peter Catholic University, 1088 Budapest, Hungary; 5 Faculty of Medicine, Semmelweis University, 1085 Budapest, Hungary; fussyp@invitel.hu (P.F.); luca.zoeldi@gmail.com (L.Z.); szonja08760@gmail.com (S.G.)

**Keywords:** gut microbiome, alpha diversity, intima-media thickness, carotid atherosclerosis, ultrasound

## Abstract

*Background and Objectives:* There is an increasing focus on the effect of the gut microbiome on developing atherosclerosis, but there is still no unified standpoint. We aimed to find associations between intestinal microbiome diversity and a marker of subclinical atherosclerosis, the carotid intima-media thickness (IMT). *Materials and Methods:* Recruited from the Hungarian Twin Registry, 108 monozygotic (MZ) twins (mean age 52.4 ± 14.1 years, 58% female) underwent a comprehensive carotid ultrasound examination (Samsung RS85). Of the 108 MZ twins, 14 pairs (mean age 65 ± 6.4 years, 71% female) discordant for carotid IMT were selected to undergo a stool sample collection. A special stool sampling container was mailed and received from each participant. After DNA extraction, library construction was performed specifically for the V3–V4 hypervariable region of microbial 16S rRNA. Next, the microbiome composition of the samples was determined using Kraken software. Two hypotheses were tested with the exact permutation test: (1) in the group with normal IMT, the Shannon index of the phyla is higher; and (2) the Firmicutes/Bacteroidetes ratio is greater in the group with high IMT values. Furthermore, the abundance of different bacterial strains present at higher and normal IMT was also explored. Statistical analysis was carried out using R software. *Results:* Increased Firmicutes/Bacteroidetes ratio was associated with increased IMT (mean Firmicutes/Bacteroidetes ratio of IMT > 0.9 and IMT < 0.9 groups: 2.299 and 1.436, respectively; *p* = 0.031). In the group with normal IMT values, a substantially higher fraction of *Prevotellaceae* was observed in contrast with subjects having subclinical atherosclerosis. However, there was no significant difference in the alpha diversity between the two groups. *Conclusions:* The determining role of individual genera and their proportions in the development and progression of atherosclerosis can be assumed. Further studies are needed to clarify if these findings can be used as potential therapeutic targets.

## 1. Introduction

Carotid artery atherosclerosis (CAS) is known to be associated with increased risk for cardiovascular diseases (CVDs) [1,2,3], which are the leading causes of death globally, taking an estimated nearly 18 million lives each year [4]. As a cardiovascular marker, carotid intima-media thickness (IMT) has been more widely studied, and underlying genetic vs. environmental factors have been investigated. Zhao et al. found that genetic factors had a significant effect on carotid IMT variation [5]. Age, systolic blood pressure, and high-density lipoprotein (HDL) have also been shown to be significantly associated with carotid IMT. Segment-specific genetic influence of carotid IMT was studied in a large Korean twin population, which reported moderately high heritability of 0.48 for common, 0.38 for carotid bifurcation, and 0.45 for internal carotid artery [6]. Well-known cardiovascular risk factors associated with carotid IMT, influencing specific segments of the carotid artery, are, for men, alcohol consumption (bifurcation), physical activity (common carotid artery (CCA) and internal carotid artery (ICA)), body mass index (BMI) (all segments), diabetes (bifurcation and ICA), hypertension (ICA), and HDL cholesterol (CCA and bifurcation); on the other hand, for women, they are smoking (bifurcation), hypertension (CCA), total and low-density lipoprotein (LDL) cholesterol (bifurcation and ICA), and high-sensitivity C-reactive protein (hs-CRP) (CCA and ICA) [6]. These findings explain the differences in the incidence of cardiovascular disease between men and women. Although genetic variation and environmental risk factors are known to influence carotid atherosclerosis, recent research has shown that the composition and diversity of the gastrointestinal microbiome also affect the development of CVDs [7,8,9].

The microbiome is a vast and complex polymicrobial ecosystem that co-exists with the human body and plays a significant role in shaping the immunological phenotypes of the host. In human and animal models, alterations in intestinal microbiome diversity change in parallel with lifestyle (e.g., smoking, dietary preferences, and physical exercise) [10].

A large metagenome-based study revealed several microbial pathways associated with CVD risk that proved to be largely independent of diet and inflammation, further reinforcing efforts to introduce microbiome-targeted therapy for prevention and treatment [11]. Infection, immunity, and the association of bacterial products with the development of atherosclerosis such as immune activators or diet-related metabolites, especially the new microbiome-dependent dietary metabolite, trimethylamine N-oxide (TMAO), also play a central role. Understanding the gut microbiome mechanism will help advance the treatment of atherosclerosis [12,13,14]. The specific components metabolized by nutrition and the intestinal microbiome can have a variety of effects on atherosclerosis; for example, dietary fiber is preferred, while the bacterial TMAO metabolite is considered harmful [15,16].

The importance of some species in CVDs has been also demonstrated [17]. Despite the contradictory results [18], increased Firmicutes/Bacteroidetes ratio—the two dominant bacterial phyla that represent more than 90% of the total community [19]—is frequently cited as a hallmark of obesity, hypertension, and microbiome dysbiosis [20,21,22]. In the Moscow Study, different metabolic changes were associated with the different abundance of genera in cases of cardiovascular risk factors: obesity—higher *Serratia* and *Prevotella*; abdominal obesity—higher *Serratia* and *Prevotella* and lower *Oscillospira*; glucose metabolism disturbances—higher *Blautia* and *Serratia*; arterial hypertension—high *Blautia.* IMT was also higher in the cluster with lower diversity [23]. In a Swedish study, the genus *Collinsella* was enriched in patients with symptomatic atherosclerosis, whereas *Roseburia* and *Eubacterium* were enriched in healthy controls [24]. A protective property of *Akkermansia muciniphila* against atherosclerosis has also been explored [25]. Increased abundance of *Enterobacteriaceae* and *Streptococcus* spp. was found in patients with atherosclerotic CVD [26].

Although the association of specific bacterial taxa with atherosclerosis is already known, many questions remain unanswered to fully understand how the microbiome contributes to atherosclerosis and CVD. By studying monozygotic (MZ) twins—since their genome is nearly the same—the genetic factors can be mostly ruled out; therefore, the role of common and unique environmental factors can be explored.

Our study aimed to explore the relationship between gut microbiome diversity and one of the most commonly investigated radiological markers of subclinical atherosclerosis, the carotid IMT. In MZ twins, we examined the number of different bacterial strains present at higher and normal IMT. Furthermore, we tested two hypotheses in discordant MZ pairs: (1) in the group with normal IMT, whether the Shannon index of the phyla is higher; and (2) whether the Firmicutes/Bacteroidetes ratio is greater in the subjects with high IMT values.

## 2. Materials and Methods

### 2.1. Study Participants

Between October 2018 and April 2020, 108 asymptomatic MZ Hungarian twins (54 pairs, mean age 52.4 ± 14.1 years, 58% female) recruited from the Hungarian Twin Registry [27,28] underwent comprehensive carotid ultrasound examination. Exclusion criteria were pregnancy, previous carotid surgery, acute infection in the past 3 weeks before the study, and underlying oncologic disease.

Blood pressure values were measured by TensioMed Arteriograph (Medexpert Ltd., Budapest, Hungary), and BMI was calculated (OMRON Ltd., Kyoto, Japan). Questionnaires were completed to assess general health status and medical history.

The mean and maximal IMT on the left and right common carotid artery was measured. MZ discordance was defined as one individual of a twin pair having a maximal carotid IMT >0.9 mm, and the other twin having a maximal carotid IMT < 0.9 mm on either the left or right or both sides. In total, 14 discordant MZ pairs (*n* = 28, aged 52–73 years, mean age 65 ± 6.4 years, 71% female) meeting these criteria were found and analyzed.

All subjects gave their informed consent for inclusion before they participated in the study. The study was conducted in accordance with the Declaration of Helsinki, and the protocol was approved by the Semmelweis University Regional and Institutional Committee of Science and Research Ethics (SE TUKEB 189/2014, amendment on 10 October, 2016, and 7 December, 2018).

### 2.2. Carotid Ultrasound

The ultrasound examination was performed with the Samsung RS85 device [29] and a high-resolution linear LM4-15B (15 MHz) transducer. The vessels were traced from the proximal origin of CCA to the bifurcation. Longitudinal recordings were saved for CCA proximal, middle tertiary and a distal section, and bifurcation. Automated IMT measurements were performed on the CCA distal section at a distance of 0.5–2 cm from the bifurcation with the Arterial Analysis program on the distal wall, and an average value was used in the analysis. According to the 2018 ESC/ESH Guidelines for the management of arterial hypertension, carotid IMT >0.9 mm was determined as abnormal [30].

### 2.3. Sample Collection and Processing

Of the 108 MZ twins, 14 pairs (mean age 65.0 ± 6.4 years, 71% female) discordant for carotid IMT were selected to undergo a stool sample collection.

Twin pairs were requested to mail their stool sample in the fecal sampling container, specially developed for this purpose after proper packaging, the same day when the sample was collected or the next day at the latest. Stool samples received within a few days of posting were processed and evaluated in cooperation with the Institute of Medical Microbiology, Semmelweis University.

After DNA extraction, library construction was performed specifically for the hypervariable region V3–V4 of microbial 16S rRNA according to the protocol recommended by Illumina and preferred in microbiome studies [31]. Libraries labeled with individual index pairs and validated with an Agilent 2100 Bioanalyzer were sequenced after pooling on an Illumina MiSeq platform by running a 600-cycle MiSeq Reagent Kit v3.

### 2.4. Bioinformatics and Statistical Analysis

The bioinformatics analysis of the 16S sequencing data was essentially carried out as described previously [32]. Briefly, the quality of raw reads was assessed with FastQC and MultiQC [33], the low-quality sequences were filtered and trimmed by Trimmomatic [34], and only sequences with a minimal length of 50 were kept. The low-quality and the first 12 base calls were discarded (Phred score <  20, sliding window size = 5). The read classification was performed with the Kraken2 [35,36], with k-mer size 31 against the SSU Ref NR 99 database (release 132) of SILVA [37]. Finally, the microbiome composition and the taxa abundances were estimated by the Bracken [38].

Statistical analysis was carried out using R version 3.6.3. The significance level was set at *p* < 0.05.

## 3. Results

Basic patient characteristics data (e.g., mean BMI, blood pressure, IMT; atherosclerosis risk factors) in the two groups are shown in Table 1.

### 3.1. Alpha Diversities of Discordant Twin Pairs

We hypothesized that in the group with IMT < 0.9 mm, the Shannon index of the phyla would be higher than in the group with IMT > 0.9 mm. A one-sided exact permutation test was conducted to test the sharp null that there were no differences in the two groups. The null on a 0.05 significance level could not be rejected (*p* = 0.153) (Table 2), leading to the conclusion that there may not be a significant relationship between the IMT levels and the Shannon index of the phyla.

### 3.2. Firmicutes/Bacteroidetes Ratio

We tested another hypothesis, which was that the ratio of Firmicutes/Bacteroidetes would be greater in the group with high IMT values than in the group with low IMT values. The sharp null that there is no difference between the two groups could be rejected using an exact permutation test (*p* = 0.031) (Table 2), supporting that higher Firmicutes/Bacteroidetes ratio is connected to atherosclerotic phenotype.

### 3.3. Microbial Compositions of Discordant Twin Pairs

**Figure 1:**
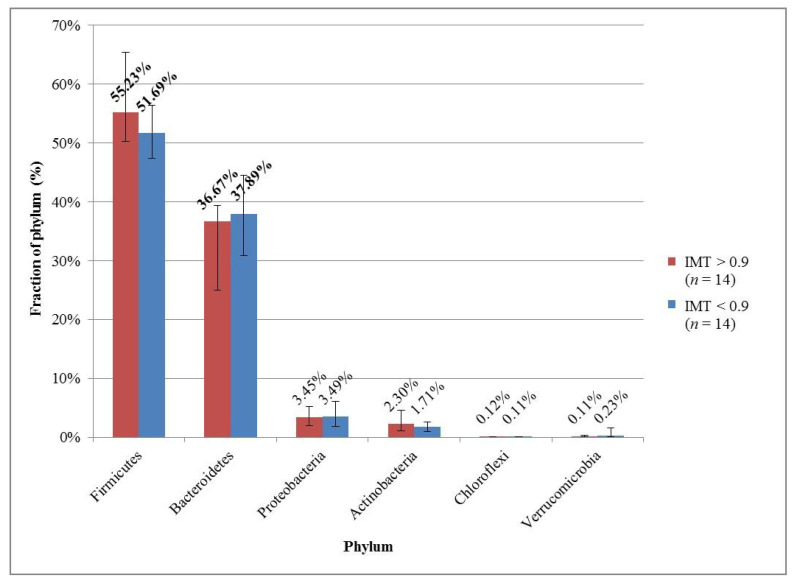
The 5 most common phyla in the groups with high and normal IMT. The descending order was based on the group with high IMT. The most prominent phyla are marked with bold font.

**Figure 2:**
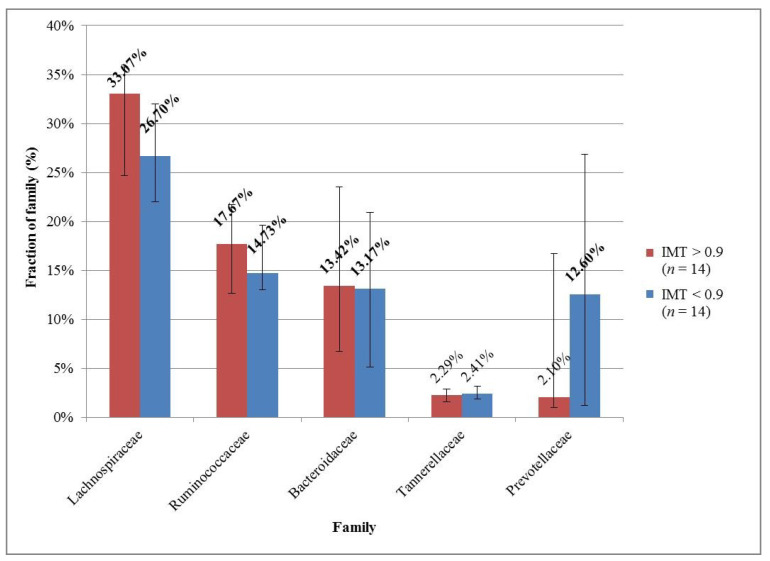
The 5 most common families in the groups with high and normal IMT. The descending order was based on the group with high IMT. The most prominent families are marked with bold font.

**Figure 3:**
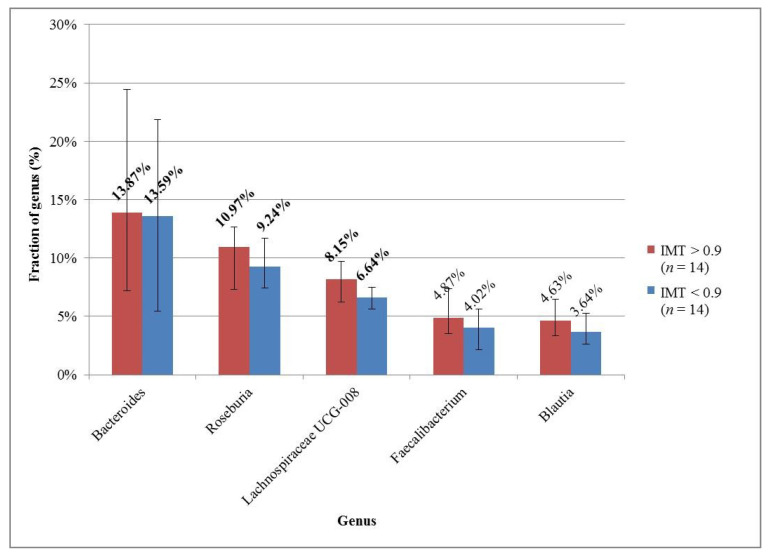
The 5 most common genera in the groups with high and normal IMT. The descending order was based on the group with high IMT. The most prominent genera are marked with bold font.

The microbial compositions in each group were also examined at 3 taxonomic levels. In both groups, the median and the interquartile range of the microbe’s fractions were counted; the results were sorted and plotted for the 5 most common microbes in each group for each taxonomy level (Figure 1, Figure 2 and Figure 3).

Although similar results were obtained in the 2 groups, greater or lesser differences were also observed.

In both groups, Firmicutes, Bacteroidetes, and Proteobacteria proved to be the most common phyla (Figure 1). Firmicutes was represented with a higher fraction in the group with high IMT values, and Bacteroidetes in second place vice versa.

At the family taxonomic level, in both groups, Lachnospiraceae, Ruminococcaceae, and Bacteroidaceae were in the top 3 (Figure 2). In the group with normal IMT values, a substantially higher fraction of Prevotellaceae could be observed compared to the group with high IMT values.

In both groups, the five most decisive genera were the same: *Bacteroides*, *Roseburia*, *Lachnospiraceae*, *Faecalibacterium*, and *Blautia* (Figure 3).

## 4. Discussion

This is the first twin study to investigate the impact of the gut microbiome on carotid IMT in discordant twins. We first report in the literature an increased Firmicutes/Bacteroidetes ratio in subjects with increased carotid IMT. Normal carotid IMT values were associated with a substantially higher fraction of Prevotellaceae. There was no significant difference in the alpha diversity between the two groups.

Although carotid IMT is an increasingly used CVD marker in microbiome studies as well, the methodology by which the data are obtained is crucial. With the advent and dynamic development of artificial intelligence, more accurate automatic measurement options are available, such as real-time correction even during the measurement [23]. Often, the IMT values measured manually by the examiner are averaged, and there is an example of post-IT analysis when the raw image material is evaluated retrospectively [39]. Using the Arterial Analysis program on the Samsung RS85 ultrasound machine, artificial intelligence technology was applied in our microbiome study.

Gut microbiota affects hypertension and atherosclerosis through many pathways [40]. This association was studied in 617 middle-aged women from the TwinsUK cohort [41] where another atherosclerotic phenotype, the carotid-femoral pulse wave velocity (PWV), a measure of arterial stiffness, was studied, and its association with the gut microbiome composition and concurrent serum metabolomics data was assessed. PWV was negatively correlated with gut microbiome alpha diversity after adjustment for covariates, leading to the conclusion that gut microbiome diversity is inversely associated with arterial stiffness in women. The previous connection is also confirmed by the Moscow Study, in which enterotyping yielded two clusters differentiated by alpha-diversity, and IMT was higher in the cluster with lower diversity (adj. *p* < 0.001) [23]. In our study, we did not find an association of lower alpha-diversity with higher IMT, which can be argued by (1) the relatively low number of participants compared to other studies, and (2) the different study population: although the gender ratio was almost the same, the geographical location (Hungary vs. Moscow and Moscow Region) and the age of the participants (mean age 65 ± 6.4 vs. 52 ± 13 years, and aged 52–73 vs. 25–76 years) differed significantly [23].

In obese people, the relative proportion of Bacteroidetes was decreased in comparison with lean people [21]. Furthermore, this proportion increased with weight loss on two types of a low-calorie diet, and their Firmicutes/Bacteroidetes ratio normalized. These results were supported by other studies [42,43,44,45,46], suggesting that the alterations in the bacterial composition are generally associated with changes in the metabolic profile of the microbiota, and the Firmicutes/Bacteroidetes ratio could be a possible hallmark for obesity. In opposition, other studies did not observe any change or even reported decreased Firmicutes/Bacteroidetes ratio in obese animals and humans [47,48,49,50]. In our study, no significant difference was detectable in BMI values between the two groups, but increased Firmicutes/Bacteroidetes ratio was found in association with increased IMT, independently of BMI, further strengthening its role as a marker of atherosclerosis phenotype. In addition, we need to be cautious, because there are several lifestyle-associated factors known to affect microbiota composition (e.g., smoking, dietary preferences, and physical exercise [10]), which were not examined in the present study; therefore, the role of these factors also need to be considered when evaluating the results.

The human gut microbiota is mostly composed of two dominant bacterial phyla, Firmicutes and Bacteroidetes, which represent more than 90% of the total community, and of other subdominant phyla including Proteobacteria, Actinobacteria, and Verrucomicrobia [18,19]. This composition remains relatively unaffected by acute perturbations, as its plasticity allows it to rapidly return to its initial composition [51]. We could confirm this finding, as the most common phyla were the same in the group with normal IMT and very similar in the other group: Firmicutes, Bacteroidetes, Proteobacteria, Actinobacteria, and Verrucomicrobia vs. Chloroflexi. However, in their individual proportions, differences could be found: in line with the previous hypothesis of Firmicutes/Bacteroidetes ratio, Firmicutes was represented with a higher fraction in the group with high IMT values, and Bacteroidetes vice versa.

In scientific literature, conflicting results can be found about the association between *Prevotella* and cardiometabolic and cardiovascular disorders. In the Moscow Study, a higher abundance of *Prevotella* was significantly associated with obesity [23]; and in middle-aged, eastern Polish men with improper levels of total and LDL cholesterol values, *Prevotella* were enriched or showed an upward trend [52]. Stroke and transient ischemic attack—as consequences of atherosclerosis—patients had fewer commensal or beneficial genera including *Bacteroides*, *Prevotella* and *Faecalibacterium*, and more opportunistic pathogens, such as *Enterobacter*, *Megasphaera*, *Oscillibacter*, and *Desulfovibrio* [53]. In parallel with this, in our study, a substantially greater fraction of the Prevotellaceae family in the group with normal IMT values compared to the group with high IMT values was found.

In microbial studies, one major issue is the accurate identification of microbes constituting the microbiota, which requires compromises depending on the method. Microbiome studies have frequently utilized sequencing of the conserved 16S rRNA gene, but whole-genome shotgun sequencing (WGS) is also popular. A comparative study demonstrates that WGS has multiple advantages compared with the 16S amplicon method, including enhanced detection of bacterial species, increased detection of diversity, and increased prediction of genes [54]. In addition, increased length, either due to longer reads or the assembly of contigs, improved the accuracy of species detection. However, WGS is not widespread due to its disadvantages, such as its price and the massive volume of the dataset. Thus, 16S rRNA sequencing is still more commonly used with its drawbacks (e.g., high sequence similarity between related species and as a consequence, inaccuracy in the identification of individual species). We used the 16S amplicon method which is a limitation of our study compared to those where WGS could be used.

Another limitation is the relatively low number of participants (e.g., discordant MZ pairs who met the selection criteria). The sample size was influenced by the difficulty of the postal system, where, despite the shield which preserved the composition of the stool sample, shipping complications occurred. With the detailed sampling guide and the recommended postage deadline after sampling, we also tried to encourage participants to increase the quality of the incoming sample. However, in some cases, sampling had to be repeated due to inadequate quality, which limited the sample size augmentation. A further limitation of the study was the lack of blood sampling, which failed the investigation of the effect of classical atherosclerotic risk factors (e.g., cholesterol, glucose levels) on the investigated associations.

## 5. Conclusions

This is the first Hungarian twin study to assess the gut microbiome in twins. We confirmed findings of other studies in which increased Firmicutes/Bacteroidetes ratio was reported in subjects with subclinical atherosclerosis represented by increased carotid IMT. Normal carotid IMT values were associated with a substantially higher fraction of Prevotellaceae. The determining role of individual genera and their proportions needs further intensive examinations.

## Figures and Tables

**Table 1:** Basic patient characteristics data in the two groups.

Characteristic	IMT > 0.9 Group (*n* = 14)	IMT < 0.9 Group (*n* = 14)
BMI (kg/m^2^; mean ± SD)	28.3 ± 3.3	28.3 ± 3.7
Systolic blood pressure (mmHg; mean ± SD)	135.2 ± 16.6	133.2 ± 17.8
Diastolic blood pressure (mmHg; mean ± SD)	79.2 ± 9.9	78.9 ± 10.7
Carotid IMT max (mm; mean ± SD)	0.94 ± 0.16	0.81 ± 0.13
Smoking (n)	1	3
Regular coffee consumption (n)	8	8
Regular sport activities (n)	6	6
Diabetes (n)	3	2
Hypertension (n)	8	4
Dyslipidemia (n)	5	1

IMT: intima-media thickness; BMI: body mass index; SD: standard deviation.

**Table 2:** Results of exact permutation tests of Firmicutes/Bacteroidetes ratio and Shannon index of the phyla between the two groups.

Results	Mean (IMT > 0.9) (*n* = 14)	Mean (IMT < 0.9) (*n* = 14)	Mean Difference	*p* Value *
Firmicutes/Bacteroidetes ratio	2.299	1.436	0.863	0.031 (0.018, 0.047)
Shannon index of the phyla	1.35	1.44	−0.09	0.153 (0.124, 0.184)

* *p* value was estimated using 999 Monte Carlo replications, 99% confidence interval in parenthesis.

## Data Availability

Data available on request.

## Abbreviations

BMI	body mass index
CAS	carotid artery atherosclerosis
CCA	common carotid artery
CVDs	cardiovascular diseases
HDL	high-density lipoprotein
hs-CRP	high-sensitivity C-reactive protein
ICA	internal carotid artery
IMT	intima-media thickness
LDL	low-density lipoprotein
MZ	monozygotic
PWV	pulse wave velocity
TMAO	trimethylamine *N*-oxide
WGS	whole-genome shotgun sequencing
